# Ethanol exposure interacts with training conditions to influence behavioral adaptation to a negative instrumental contingency

**DOI:** 10.3389/fnbeh.2014.00220

**Published:** 2014-06-17

**Authors:** Regina A. Mangieri, Roberto U. Cofresí, Rueben A. Gonzales

**Affiliations:** Division of Pharmacology and Toxicology, College of Pharmacy, The University of Texas at AustinAustin, TX, USA

**Keywords:** ethanol, instrumental learning, habit, Long Evans rats, sucrose, omission contingency

## Abstract

We previously reported that, in male, Long Evans rats, instrumental lever pressing that had been reinforced during limited training under a variable interval (VI) schedule by oral self-administration of a 10% sucrose/10% ethanol (10S10E) solution was insensitive to devaluation of 10S10E. In contrast, lever pressing that had been reinforced under a variable ratio (VR) schedule, or by self-administration of 10% sucrose (10S) alone, was sensitive to outcome devaluation. The relative insensitivity to outcome devaluation indicated that seeking of 10S10E by the VI-trained rats had become an instrumental habit. In the present study we employed an alternative operational definition of an instrumental habit and compared the effect of reversing the action-outcome contingency on lever press performance by rats trained under the same experimental conditions. Male Long Evans rats received daily operant training, in which lever presses were reinforced by 10S10E or 10S, under VI or VR schedules. After nine sessions of VI or VR training, rats were tested over four sessions in which the instrumental contingency was changed so that a lever press would prevent reinforcer delivery for 120 s. We found that rats that had been trained to lever press for 10S10E under the VR schedule showed a greater change in lever pressing across testing sessions than those that had received 10S10E reinforcement under the VI schedule. There was no such interaction with reinforcement schedule for rats that had received only 10S reinforcement during training. These findings are consistent with those of our previous study, and provide further evidence that addition of ethanol to sucrose may promote habitual responding in an instrumental task.

## Introduction

While socially acceptable and pervasive, alcohol use is infamous for sometimes becoming a “bad habit.” Unlike goal-directed actions, which are instrumental behaviors sensitive to both the value of their outcome and the causal relationship between performance and outcome acquisition (i.e., the instrumental contingency), “habits” are contextually-elicited, well-practiced behaviors performed automatically (viz., with minimal cognitive effort) in a relatively rigid and inflexible fashion. Of particular concern is whether and under what circumstances drugs of abuse promote or accelerate behavioral automaticity, a question that can be investigated experimentally using operant self-administration paradigms. To characterize the “goal-directed” vs. “habitual” nature of instrumental behaviors, a number of investigators have employed experimental manipulations that reveal the degree to which the instrumental outcome itself is the stimulus for the behavior. For example, the canonical test for “habitual” instrumental behavior involves first, manipulating the value of the instrumental outcome (most commonly, reducing), and then observing the behavior in an operant session without reinforcer feedback (viz., in extinction). In this classical operational definition, “habitual” seeking behavior is that which shows insensitivity to outcome devaluation (Dickinson, 1985)—despite a devalued outcome, instrumental performance persists as if no manipulation had occurred. Such “insensitivity to outcome devaluation” implies that when the animal is in that particular context and the instrumental outcome is not present, a mental representation of the instrumental outcome is not the predominant factor driving instrumental performance. It is inferred, therefore, that under those conditions the behavior is executed “automatically,” without consideration of its outcome.

One method for inducing outcome devaluation is to use injections of lithium chloride (LiCl) to pair malaise with ingestion of the reinforcing substance (Adams and Dickinson, 1981). Previously, we used a single LiCl-pairing to investigate the nature of operant lever pressing that either had been reinforced during training sessions only by a sucrose solution (10% w/v, 10S) or that had been reinforced by both 10S and by a sucrose solution containing ethanol (10% w/v: 10% v/v, 10S10E) (Mangieri et al., 2012); for other examples of LiCl devaluation of ethanol see Dickinson et al. (2002); Samson et al. (2004). When evaluated after seven to nine operant self-administration sessions, seeking behavior by male Long-Evans rats trained to press a lever for 10S10E on variable interval (VI) schedules of reinforcement was unaffected in the test session following LiCl-pairing with 10S10E. In contrast, lever pressing in the test session by rats trained to self-administer 10S10E on variable ratio (VR) schedules was decreased following pairing of LiCl with 10S10E, as was that by rats trained to self-administer 10S on VI schedules following pairing of LiCl with 10S. Thus, by the classical operational definition, it could be argued that even a limited history of reinforcement with alcohol under a VI schedule can promote “habitual” alcohol seeking.

One caveat to such an interpretation is that when the paired outcome is a mixed substance consisting of components with distinct psychopharmacological and sensory properties, such as a sweetened alcohol drinking solution, there is a degree of uncertainty regarding the specificity of devaluation that clouds interpretation. Specifically, LiCl pairing could devalue the rewarding taste of sucrose, the caloric contribution of sucrose and ethanol to reward, or the rewarding effect of ethanol intoxication, and it is difficult to parse out which component was devalued by the LiCl pairing procedure. In view of this uncertainty, an independent measure of the degree of habit formation that occurs with 10S and 10S10E solutions will be helpful to confirm or refute the interpretation of the previous experiment. Thus, in order to clarify interpretation of our previous findings, we employed here an assay for the nature of instrumental behavior that does not manipulate value of the outcome, but rather the instrumental contingency (Dickinson et al., 1998; Yin et al., 2006; Coutureau et al., 2012; Fanelli et al., 2013; Shillinglaw et al., 2014).

In the first experiment presented here, we trained male Long-Evans rats to press a lever for the opportunity to orally self-administer an ethanol-sucrose drinking solution using the same limited training protocols as in our previous work (Mangieri et al., 2012). We then observed how lever pressing changed across four test sessions in which receipt of the desired outcome (access to the drinking solution) was contingent upon omission of the previously reinforced instrumental behavior. It is well-established that both the schedule of reinforcement used during instrumental training, and the amount of training, are critical factors in the development of “habitual” responding (see Yin and Knowlton, 2006, for review). On the basis of the literature, as well as our own work, we expected that, when tested after only limited instrumental training, lever pressing by VI-trained 10S10E-seeking rats would be less affected by the new, negative contingency than that of VR-trained 10S10E seeking rats. In order to determine if the same VI training schedule would similarly drive habitual performance for rats that were never exposed to ethanol, we performed a second experiment in which the two groups of rats were trained with only 10S reinforcement.

## Materials and methods

### Animals

All experimental procedures were approved by the Institutional Animal Care and Use Committee of the University of Texas at Austin (current Animal Use Protocol #2011-00069) and performed as per the Guidelines for the Care and Use of Animals in Neuroscience issued by the National Academies. Naïve, male Long Evans rats (*N* = 26) weighing 200–225 g upon arrival from Charles River Laboratories (facility P04) were maintained on a 12-h light/dark cycle in a temperature-controlled room (72 ± 4°F) at the University of Texas at Austin Animal Resources Center with *ad libitum* access to standard chow (LabDiet® 5LL2, PMI Nutrition International, Richmond, IN) and water except for as described under section Behavioral Training and Testing. Rats were weighed prior to any procedures, all of which took place during the light phase.

Upon arrival, group housed rats (three per cage) were allowed 1 week of habituation to their new environment. During this week only, the experimenter handled rats daily (approximately 10 min per rat). Two days before commencing behavioral training, rats were separated into individual cages, remaining individually housed and receiving no additional handling, except as required for operant chamber entry and exit, throughout the experiment. A total of four cohorts were trained for these experiments: two 10S10E (18 months apart) and two 10S (1 month apart).

### Apparatus and drinking solutions

As in Mangieri et al. (2012), instrumental training sessions took place within operant conditioning chambers (30.5 × 24.1 × 21 cm interior) housed inside sound-attenuating cubicles in a dedicated room (chambers and cubicles from Med Associates, Inc., Georgia, VT). Cubicles lacked exterior doors, but were equipped with exhaust fans to provide white noise during all sessions. For the duration of any session, a house light located at the top center of the left wall remained lit and a 4.6 cm-wide retractable lever located along the distal portion of the right wall remained inserted into the chamber 6.35 cm above the metal bar flooring. A retractable bottle assembly located on the outside of the proximal right chamber wall held a bottle containing the drinking solution. All chamber components were controlled by Med-PC IV software (Med Associates, Inc., Georgia, VT). Upon earning reinforcement (as determined by the program), the bottle's metal sipper tube was inserted into the chamber and then retracted 10 s later, allowing the rat brief access to the drinking solution. Operant chambers were also equipped with a lickometer circuit.

The drinking solutions, 10% sucrose (w/v) and 10% sucrose (w/v): 10% ethanol (v/v), were prepared approximately every 3 days from ultra-pure sucrose (MP Biomedicals, Solon, OH), 95% ethanol (AAPER Alcohol and Chemical Co., Shelbyville, KY), and distilled water, and stored at 4°C.

### Behavioral training and testing

#### Shaping

Water deprivation began 22 h prior to the first session in the operant chamber, after which water was available in the home cage for 2 h. During this session only, the sipper tube was inserted into the chamber for the entire 20-min duration. Lever presses were inconsequential, but 20 mL of 10S was available for *ad libitum* consumption. Approximately 24 h later, water deprived rats received an operant conditioning session in which lever pressing behavior was shaped using a fixed ratio 1 (FR1) schedule: the sipper tube, containing 10S, was inserted for 10 s following any lever press. Shaping involved encouraging the rat to explore the inserted lever by wetting it with a sucrose-soaked cotton swab at the start of the session. When necessary, rats were reminded of the lever's or sipper tube's location by tapping on the adjacent plexiglass wall. Occasionally, it was necessary to remind rats of the lever's operability by causing it to move up and down while in the animal's line of sight. This shaping session lasted 20 min minimum and 40 min maximum depending on whether the rat appeared to be on the verge of learning the instrumental contingency. A rat advanced onto Training if it earned and engaged approximately 15–20 sipper tube deliveries during the shaping session without experimenter assistance. A minority of rats required a second session, but all rats acquired instrumental lever-pressing behavior within 2 days.

#### Training

**Figure 1:**
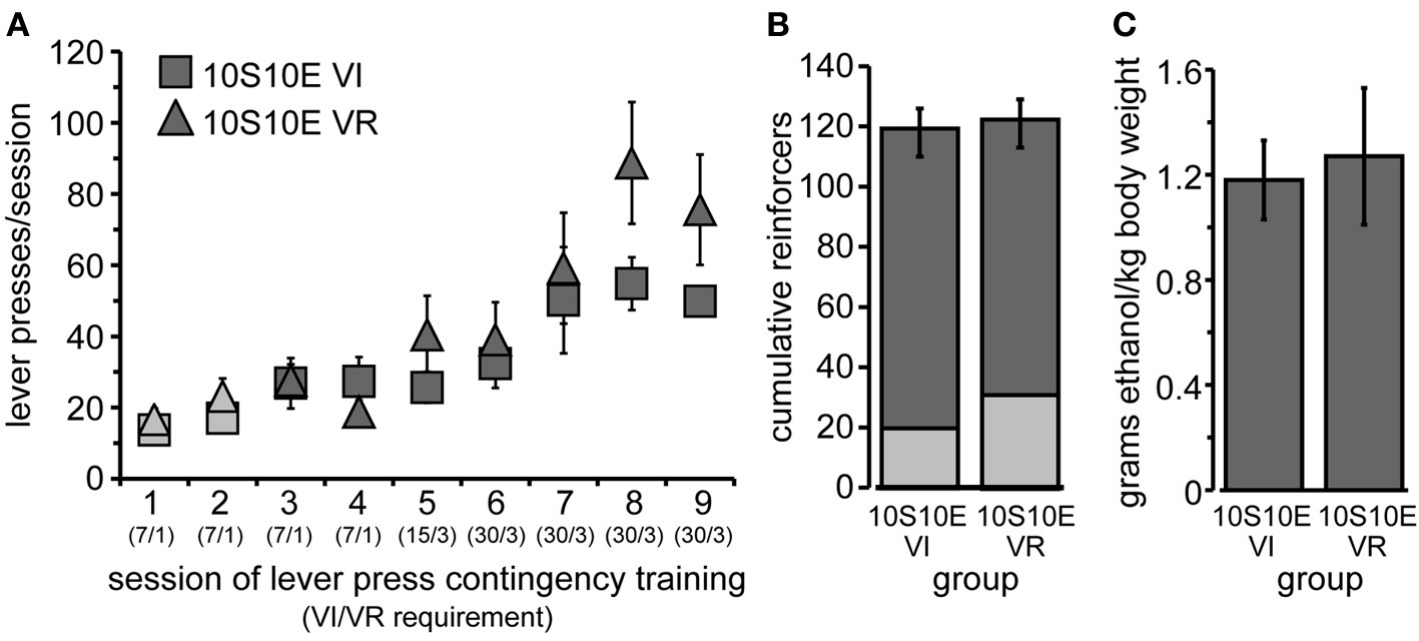
**Training of 10S10E groups. (A)** Lever pressing increased similarly across training sessions for the two groups, 10S10E VI (squares) and 10S10E VR (triangles). Symbols for 10S-reinforced sessions are filled with lighter gray; those for 10S10E-reinforced sessions are darker gray. The numbers in parentheses beneath the session numbers on the x-axis indicate the programmed reinforcement criteria in effect for each group during that session (average time interval, in seconds, before a press yielded reinforcer delivery for the VI group/average number of lever presses required by VR group for reinforcer delivery). **(B)** The two groups did not differ in the cumulative number of reinforcers received during 10S-reinforced sessions (lighter gray portion of bar), 10S10E-reinforced sessions (darker gray portion), or all sessions combined (entire bar). **(C)** The dose of ethanol self-administered in a single operant session (shown here for the last training session) was similar between the two groups.

Upon successful acquisition of the lever-press response, rats were no longer water deprived, and began instrumental training, receiving one 20-min operant session per day, 4–5 days per week. All rats completed two sessions with 10S, followed by seven with 10S10E or 10S, for a total of nine training sessions. An ethanol dose of at least 0.3 g/kg on the last three training sessions was required *a priori* for 10S10E-reinforced rats before advancing onto Testing. Rats completed either a VI or VR training protocol. The progression of reinforcement schedules and drinking solutions is depicted in Figure 1A (10S10E groups) or Figure 4A (10S groups). Med-PC programs were written such that upon earning a reinforcer delivery, any newly selected interval or ratio response requirement entered into effect only after the 10-s sipper tube access period terminated. In order minimize differences in overall reinforcement between VI- and VR-trained groups, a maximum of 25 reinforcer deliveries was allowed per session. After the 25th reinforcer delivery, the operant session was terminated by retraction of the lever and sipper tube, but the rat remained in the chamber until 20 min had elapsed from the beginning of the session.

#### Testing

Following training, the flexibility of seeking behavior was tested using four 20-min operant sessions of an omission interval (OI) schedule of reinforcement: access to the drinking solution was granted every 120 s unless the rat pressed the lever, resetting the timer on reinforcer delivery. The selection of the 120 s interval was based in part on the finding that this interval was at least eight times greater than the longest median inter-response interval observed in any of the groups tested on the last day of training. Although it was possible to earn nine instances of drinking solution access per OI session, no rat earned the maximum on any of the four sessions. Breaks (days without an operant session) during the testing phase differed between 10S10E, but not 10S cohorts. For the groups trained to drink 10S10E there were 3 separate patterns for “days off” before and during the omission test sessions: (1) 1 day off before session three and 1 day off before session four (*n* = 7); (2) 2 days off before session one and 1 day off before session four (*n* = 6); and (3) one rat had 1 day off before session one and 2 days off before session four. For the 10S groups all rats had “break” pattern (2) described above.

### Data collection, representation, and analysis

Med-PC IV software recorded the occurrence and time of each lever press, insertion of the sipper tube (reinforcer delivery), and lickometer circuit completion (contact with the sipper tube) in the session. At the end of every operant session, the remaining volume of drinking solution was manually measured and recorded. A plastic tray placed under the bottle assembly collected leaked fluid, which was added to the volume remaining in the bottle. Estimates of ethanol consumption (g/kg) were corrected for spillage.

Graphical representations of data (created with Microsoft Excel and Adobe Illustrator) show group means for the indicated measure; error bars indicate the standard error of the mean (s.e.m.). Data were analyzed using SPSS (version 17.0, IBM) univariate or repeated measures general linear model procedures, as appropriate. Specifically, repeated measures ANOVA with both between-groups and within-subjects factors was used to analyze training and testing behavior across time. Significant (*p* < 0.05) group (schedule) × time (session or bin) interaction effects were further investigated by analyzing the simple effect of session (or bin) for each group. For simple effects tests the *F*-value and significance were computed using the MS_error_ and df taken from the overall analysis (Kirk, 1982). A simple effect of time was considered significant if *p* was < 0.025 (Bonferroni correction), and for subsequent *post-hoc* comparisons, we also applied the Bonferroni correction to maintain a Type I error rate <0.05 for each group of tests. Effect sizes for the within-between interaction in the overall ANOVA were obtained using SPSS and G^*^Power 3.1.9.2 (Faul et al., 2007). Change in lever pressing across test sessions was also compared for the 10S10E VI and VR groups by fitting the mean lever presses per session to either a linear or quadratic function. An *F*-test was used to compare which of the functions better fit the data (Kenakin, 1997).

Data from the 10S10E groups were analyzed separately from the 10S groups. 10S cohorts were trained a month apart and 19–20 months after the first 10S10E cohort, and 1–2 months after the second. Of the 26 rats used in this experiment, two were excluded from statistical analyses: one due to freezing behavior during the first test (OI) session, the other due to equipment malfunction during the third test (OI) session.

## Results

### Experiment 1

#### Training

After the initial acquisition of operant lever pressing, rats were assigned to one of two groups, “10S10E VI” (*n* = 6) or “10S10E VR” (*n* = 7). Body weights prior to the 1st training session did not differ (mean ± s.e.m. grams: *VI* = 293 ± 4; *VR* = 297 ± 8). Lever pressing escalated similarly across the nine training sessions for the two groups [Figure 1A; main effect of group: *F*_(1, 11)_ = 0.99, ns; main effect of session: *F*_(8, 88)_ = 17.57, *p* < 0.001; session × group interaction: *F*_(8, 88)_ = 1.83, n.s.]. The lack of a significant main effect of group or interaction between group and session indicates that the response rates were matched between the groups including the last training session. We also looked at the frequency distribution of the inter-response intervals for the VI and VR groups on the last day of training, and both distributions tailed off to near zero after an interval of >60 s (VR median = 2.1 s; VI median = 14.0 s). Likewise, the number of reinforcers received per session increased similarly for the two groups over time [data not shown; main effect of session: *F*_(8, 88)_ = 2.64, *p* = 0.01; session × group interaction: *F*_(8, 88)_ = 1.68, n.s.]. We also assessed cumulative reinforcement (represented in Figure 1B). There was no difference between the two groups in the total number of reinforcers received over all nine training sessions [*F*_(1, 11)_ = 0.02, n.s.], nor in the number of reinforcers received during the 10S sessions [*F*_(1, 11)_ = 1.94, n.s.] or the 10S10E sessions [*F*_(1, 11)_ = 0.15, n.s.]. Finally, we confirmed that earning equivalent numbers of 10S10E reinforcers corresponded to administering equivalent doses of ethanol (shown for the final training session by Figure 1C). Importantly, ethanol doses (g/kg) self-administered by the end of training were intoxicating (collapsed group means ± s.e.m. for the last three training sessions: 0.99 ± 0.13, 1.18 ± 0.15, 1.23 ± 0.15). Unpublished work from our lab shows that intake of approximately 1 g/kg ethanol in Long Evans rats produces an average blood alcohol concentration of 46 mg/dl. This concentration has been found to produce signs of intoxication in humans (Brasser et al., 2004), and the dose consumed has been found to produce discriminative stimulus effects of ethanol (Quertemont et al., 2003).

#### Testing

**Figure 2:**
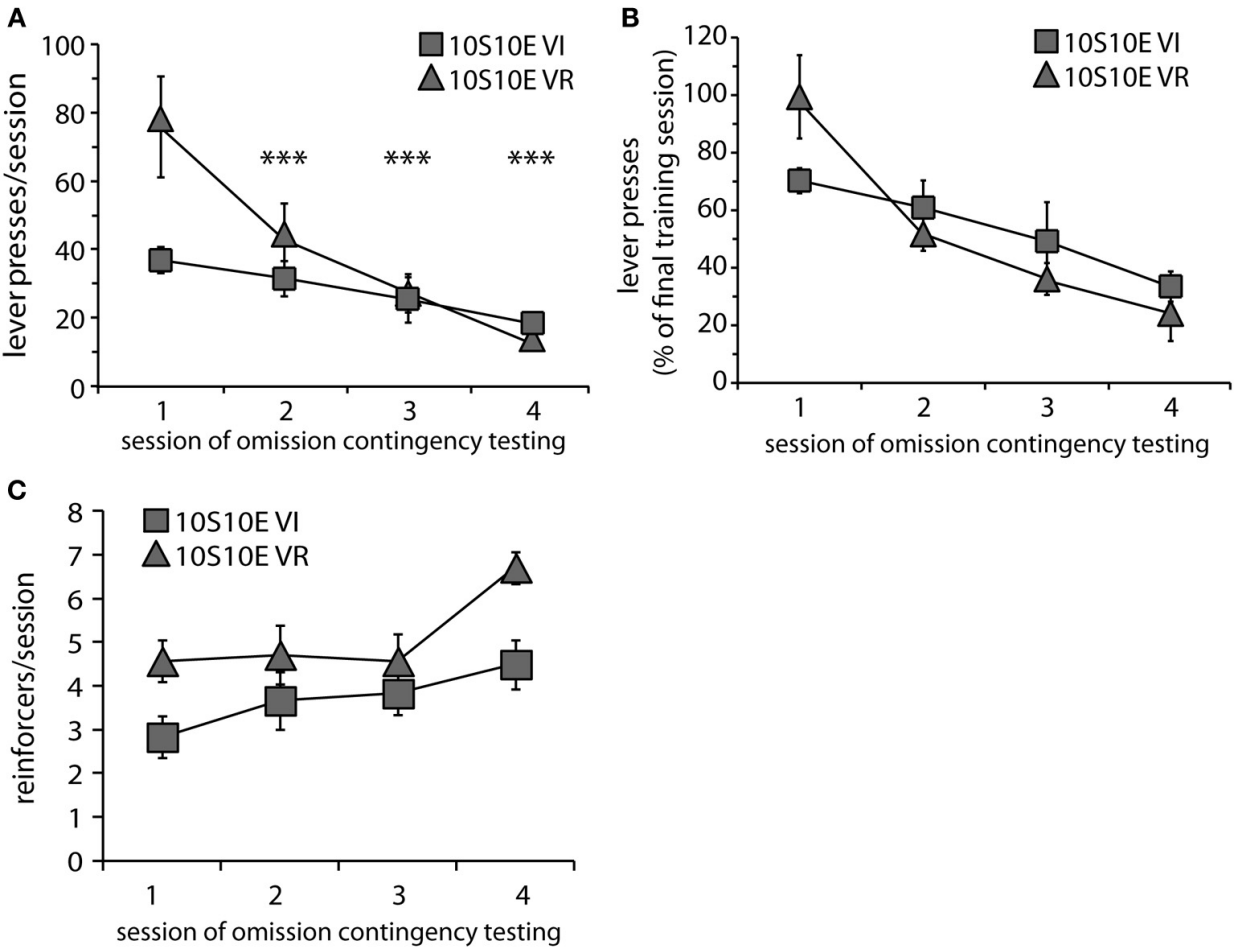
**Testing of 10S10E groups. (A)** The number of lever presses per session declined across testing for the 10S10E VR (triangles), but not the 10S10E VI (squares) group. ^***^Indicates for the VR group the sessions that were significantly different from session 1 (*p* < 0.001 level of significance for *post-hoc* contrasts). **(B)** The different patterns of behavioral adaptation were still apparent when test performance was expressed relative to training performance. **(C)** The number of reinforcers received per session increased similarly for the two groups across testing, but the 10S10E VR group received more reinforcers overall.

At the end of the training period, both groups were tested over four sessions with the omission contingency in effect (omission of lever pressing for 120 s earned one reinforcer). Body weights prior to the 1st testing session did not differ (mean ± s.e.m. grams: *VI* = 388 ± 8; *VR* = 396 ± 11). Behavioral adaptation across testing is presented in Figure 2. Responses are shown both as the raw number of presses for each test session (Figure 2A) and as a percentage of the number of presses made during the last training session (Figure 2B). Two-way repeated measures ANOVA conducted on the raw data revealed that, overall, responding decayed across sessions, but the rate of decay was not the same for both groups [main effect of session: *F*_(3, 33)_ = 14.6, *p* < 0.0001; main effect of group: *F*_(1, 11)_ = 2.0, n.s.; session × group interaction: *F*_(3, 33)_ = 4.5, *p* = 0.009]. Investigation of the simple effect of session within each group revealed that the number of lever presses per session decreased over the four test sessions for the 10S10E VR group, but not the 10S10E VI group [simple effect of session, 10S10E VR: *F*_(3, 33)_ = 19.1, *p* < 0.001; 10S10E VI: *F*_(3, 33)_ = 1.4, n.s.]. Individual *post-hoc* contrasts are shown on Figure 2A and described in the figure caption. We also analyzed the rate of response suppression for the two groups by using linear or quadratic fits to the mean responses across test sessions. The functions that best fit the data were linear for the VI group and quadratic for the VR group. These fits were significantly better than fitting both groups to a linear function [*F*_(1, 3)_ = 15.9, *p* < 0.05]. Furthermore, the VI group alone exhibited a significant linear trend in the decay of responses over sessions (*r*^2^ = 0.99, *p* < 0.05). Figure 2C shows the number of reinforcers delivered per session for each of the omission test sessions. The overall reduction of lever pressing by the two groups across testing was reflected in an increase in reinforcers earned over the four sessions of omission testing [main effect of session: *F*_(3, 33)_ = 7.2, *p* < 0.01]. The differential adaptation of lever pressing behavior between the VI and VR groups was manifested in an overall group difference in the number of reinforcers received per session [main effect of group: *F*_(1, 11)_ = 6.3, *p* < 0.05], but there was no group × session interaction [*F*_(3, 33)_ = 1.2, n.s.].

**Figure 3:**
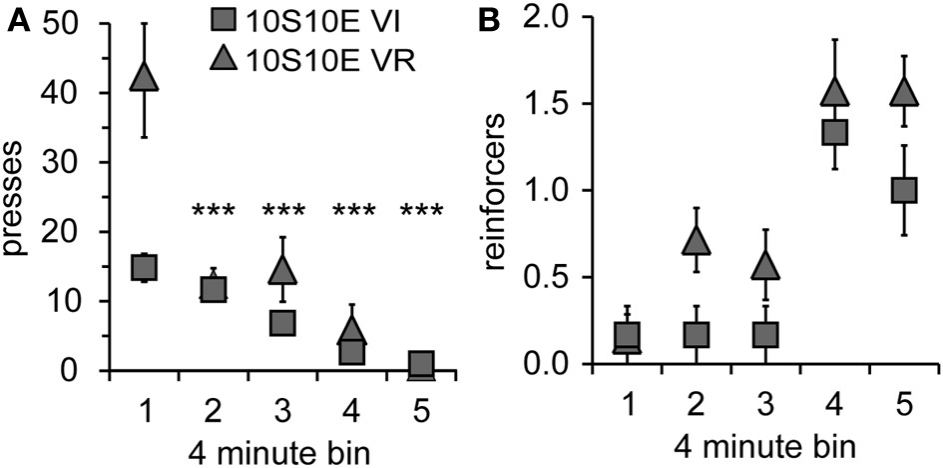
**Performance of 10S10E groups during first test session. (A)** The number of lever presses per four-minute bin decreased over the session for the 10S10E VR (triangles), but not the 10S10E VI (squares) group. ^***^Indicates for the VR group the bins that were significantly different from bin 1 (*p* < 0.001 level of significance). **(B)** The number of reinforcers received per four-minute bin increased for both groups over the session, but the 10S10E VR group received more reinforcers than the VI.

In order to gain further insight as to how the two groups were affected by the introduction of the omission contingency, we analyzed presses and reinforcers (in 4-min bins) within just the first test session. As shown by Figure 3A, both the total number and pattern of presses were different between groups [main effect of group: *F*_(1, 11)_ = 5.6, *p* = 0.038; main effect of bin: *F*_(4, 44)_ = 19.8, *p* < 0.001; bin × group interaction: *F*_(4, 44)_ = 5.7, *p* = 0.001]. Simple effects analyses revealed a significant change in lever pressing across bins for the VR, but not the VI group [10S10E VR: *F*_(4, 44)_ = 24.4, *p* < 0.001, individual *post-hoc* comparisons are shown on the figure; 10S10E VI: *F*_(4, 44)_ = 2.7, n.s.]. Figure 3B shows for each group the number of reinforcers delivered per 4-min bin across the session. Statistical analysis of these data revealed a main effect of group [*F*_(1, 11)_ = 6.5, *p* = 0.027] and bin [*F*_(4, 44)_ = 16.4, *p* < 0.001], but no bin × group interaction [*F*_(4, 44)_ = 0.7, n.s.]. Together, these analyses show that although the total number of presses and the initial rate of pressing was greater for the VR group, this group adapted to the omission contingency in such a way that it received more reinforcers during the first test session.

### Experiment 2

#### Training

**Figure 4:**
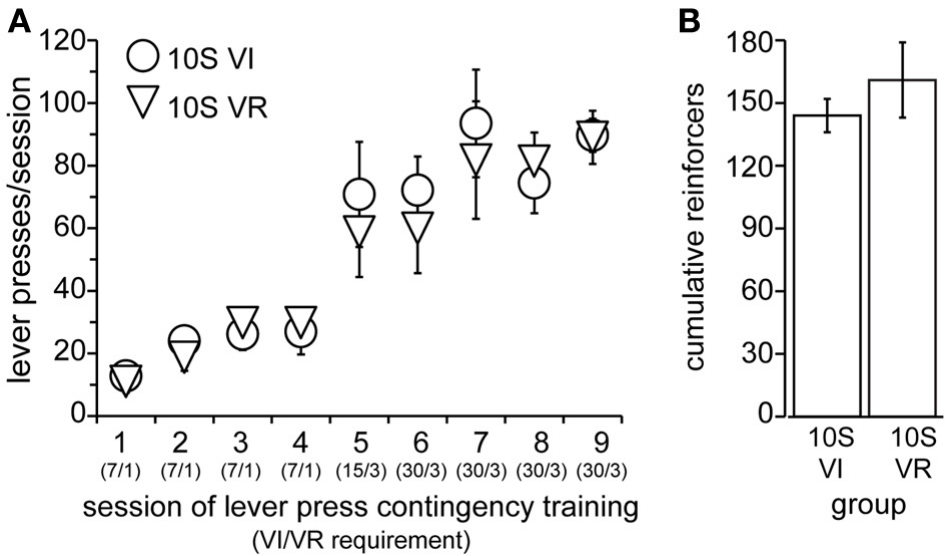
**Training of 10S groups. (A)** Lever pressing by 10S VI (circles) and 10S VR (triangles) groups across training sessions. The numbers in parentheses beneath the session numbers on the x-axis indicate the programmed reinforcement criteria in effect for each group during that session (average time interval, in seconds, before a press yielded reinforcer delivery for the VI group/average number of lever presses required by VR group for reinforcer delivery). **(B)** The two groups did not differ in the cumulative number of reinforcers received across all training sessions.

Lever press conditioning and training proceeded exactly as in Experiment 1, with the exception that the drinking solution contained in the sipper tube remained 10S throughout the experiment. Operant lever pressing was conditioned in all animals in this experiment within 2 days (data not shown), after which they were divided into two groups, “10S VI” (*n* = 5) and “10S VR” (*n* = 6) and training commenced. Body weights prior to the 1st training session did not differ (mean ± s.e.m. grams: *VI* = 295 ± 11, *VR* = 294 ± 8). The number of lever presses per session increased similarly for the two groups over the nine training sessions [Figure 4A, main effect of group: *F*_(1, 9)_ = 0.11, ns; main effect of session: *F*_(8, 72)_ = 27.82, *p* < 0.001; session × group interaction: *F*_(8, 72)_ = 0.40, n.s.]. Similar to the previous experiment, the response rates were matched between the two groups during training. Furthermore, the frequency distributions of the inter-response intervals on the last day of training for both the VR and VI groups were similar to each other (VR median = 1.8 s; VI median = 4.9 s). Overall, the number of reinforcers earned per session also increased across training [data not shown; main effect of session: *F*_(8, 72)_ = 6.03, *p* < 0.001]. We did not observe a main effect of group [Figure 4B; *F*_(1, 9)_ = 0.61, n.s.], but there was a session × group interaction [*F*_(8, 72)_ = 2.30, *p* = 0.03]. We investigated the source of this interaction, but found the simple effect of session to be significant in each group [10S VI: *F*_(8, 72)_ = 4.47, *p* < 0.001; 10S VR: *F*_(8, 72)_ = 3.79, *p* < 0.001], and no group difference for any individual session. Furthermore, comparison of reinforcers earned per session across the last four training sessions indicated there were no differences between sessions or groups by the end of training [main effect of session: *F*_(3, 27)_ = 2.71, n.s; main effect of group: *F*_(3, 27)_ = 1.99, n.s.; session × group interaction: *F*_(1, 9)_ = 0.34, n.s.].

#### Testing

**Figure 5:**
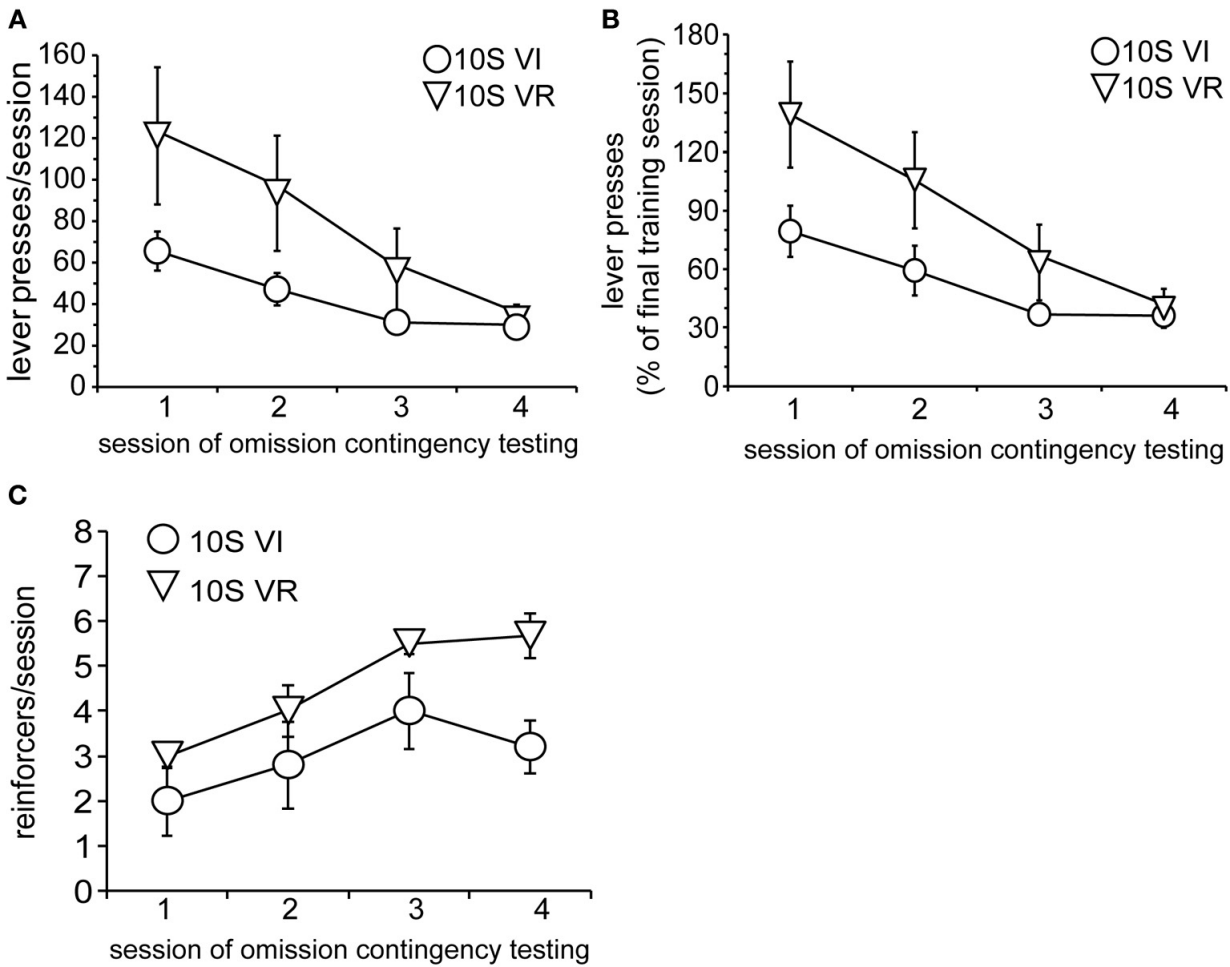
**Testing of 10S groups. (A)** Decay in the number of lever presses per session was similar for the 10S VI (circles) and 10S VR (triangles) groups across testing. **(B)** The similar patterns of behavioral adaptation were still apparent when test performance was expressed relative to training performance. **(C)** The number of reinforcers received per session increased similarly for the two groups across testing, but the 10S VR group received more reinforcers overall.

**Figure 6:**
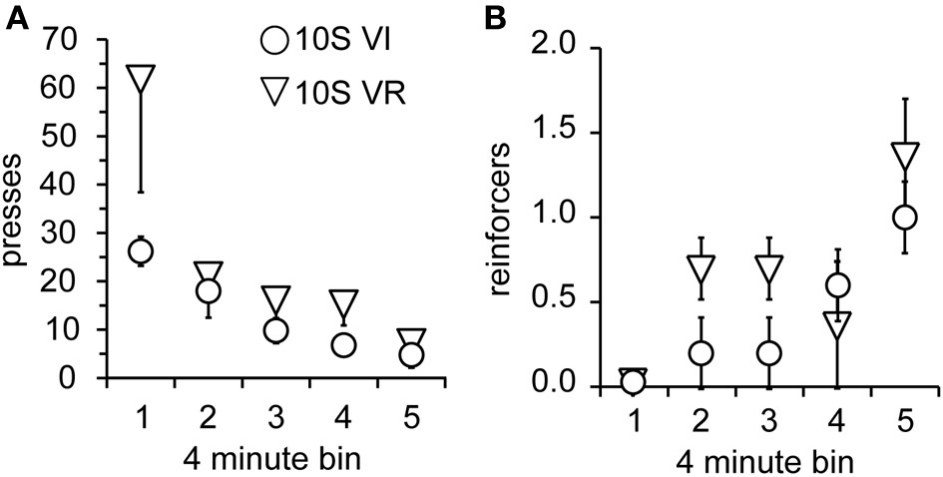
**Performance of 10S groups during first test session**. **(A)** The number of lever presses per four-minute bin decreased similarly over the session for the 10S VI (circles) and 10S VR (triangles) groups. **(B)** The number of reinforcers received per four-minute bin increased similarly over the session for the two groups.

As in Experiment 1, the two groups were tested over four sessions, during which omission of lever pressing for 120 s was necessary to receive reinforcement. Body weights prior to the 1st testing session did not differ (mean ± s.e.m. grams: *VI* = 393 ± 18; *VR* = 389 ± 15). Again, lever press performance, overall, was not different between groups and decayed across sessions [Figure 5A; main effect of group: *F*_(1, 9)_ = 2.0, n.s.; main effect of session: *F*_(3, 27)_ = 9.7, *p* < 0.001]. However, unlike the 10S10E groups in Experiment 1, there was no session × group interaction for the two 10S groups [*F*_(3, 27)_ = 1.8, n.s.]. This similarity in response decay pattern was still visually apparent when lever presses were expressed relative to the number of presses during the last training session (Figure 5B). Despite the apparent similarity of lever pressing behavior between the two groups, analysis of the number of reinforcers earned per session showed an overall group difference [Figure 5C; main effect of group: *F*_(1, 9)_ = 6.0, *p* < 0.05]. The number of reinforcers earned increased over the four sessions of omission testing [main effect of session: *F*_(3, 27)_ = 9.2, *p* < 0.0001], and this change did not interact with the overall group difference [session × group interaction: *F*_(3, 27)_ = 0.9, n.s.]. Additionally, analysis of just the first test session indicated that the number of presses [Figure 6A; main effect of bin: *F*_(4, 36)_ = 6.5, *p* < 0.001; main effect of group: *F*_(1, 9)_ = 2.2, n.s.; bin × group interaction: *F*_(4, 36)_ = 1.4, n.s.] and reinforcers per 4-min bin [Figure 6B; main effect of bin: *F*_(4, 36)_ = 7.4, *p* < 0.001; main effect of group: *F*_(1, 9)_ = 1.8, n.s.; bin × group interaction: *F*_(4, 36)_ = 1.1, n.s.] changed similarly for the two groups across the session.

## Discussion

Here we provide new evidence that ethanol can influence the transition from goal-directed to habitual expression of instrumental behavior after only 7 days of exposure during once daily operant self-administration sessions. We carried out two experiments using male Long Evans rats trained to press a lever for the opportunity to orally self-administer either ethanol-sucrose or sucrose alone under VI or VR reinforcement schedules. In both experiments, rats were first conditioned to lever press for 10S, but in the first experiment the solution changed to 10S10E after two training sessions. All other aspects of training were identical between the two experiments. In Experiment 1, the 10S10E VI and 10S10E VR groups showed no gross differences in terms of the number of lever presses or reinforcers received during training sessions. When assayed over four test sessions in which omission of lever pressing was required for the rat to receive reinforcement, the rats trained under the VR schedule showed a robust decrease in lever pressing across test sessions. In contrast, lever pressing by the 10S10E VI group changed very little across the test sessions. When we repeated this experiment with rats that received only 10S during training (i.e., that were never exposed to ethanol), the two training groups, 10S VI and 10S VR, showed a similar decrement in lever pressing across testing. We previously found that this same limited 10S10E VI, but not 10S10E VR, training protocol resulted in lever pressing that was insensitive to outcome devaluation produced by pairing LiCl-induced malaise with ethanol-induced intoxication (Mangieri et al., 2012). Together these findings are consistent with others' observations that instrumental training conditions favoring the formation of “stimulus-response (S-R) habits” (i.e., instrumental behaviors insensitive to outcome devaluation) also promote response persistence following the imposition of an omission contingency or contingency degradation (c.f. Dickinson et al., 1998; Derusso et al., 2010; Fanelli et al., 2013), although see Shillinglaw et al. (2014).

It should be noted that there are two general limitations of the present study that temper the conclusions that we can make. First, we are unable to make firm conclusions comparing the 10S10E groups and the 10S groups because the experiments were performed independently. Second, the numbers of subjects in both experiments are relatively small for behavioral experiments. It is possible that statistically significant interactions between group and session may be observed in both experiments with larger sample sizes. However, on the basis of the present results, we argue that a fundamental difference exists between the outcomes of the two experiments. Specifically, we were able to detect a significant interaction between group and session in the 10S10E groups, reflected in an effect size of 0.64 and an observed power of 0.84 for Experiment 1. On the other hand, the effect size was 0.44 and the observed power was 0.41 for Experiment 2. Therefore, we argue that, given the similar experiment sample sizes (13 vs. 11), a small effect size contributes to the lower observed power in the 10S experiment, and it would take a much larger sample size to detect a statistically significant group by session interaction, if any, when groups are trained on 10S. Clearly, this is not the case for the 10S10E experiment. Nonetheless, a full resolution of this issue would require a completely independent replication in which all factors were represented and controlled for at the same time.

Mindful of these caveats, we interpret our findings within the conceptual framework of dual decision-making/behavioral control systems. Put simply, these two, interacting, “goal-directed” and “habit,” systems learn and operate in parallel (Belin et al., 2009). Although learning occurs more rapidly *via* the goal-directed system, after some critical amount of training, the habit system becomes the dominant controller under specific conditions (Adams and Dickinson, 1981). This shift in reliance upon the goal-directed vs. habit system can be accelerated by training under interval, relative to ratio, reinforcement schedules (Dickinson et al., 1983).

We observed such an effect of reinforcement schedule in our prior work, and identified a point in training at which instrumental responding by 10S10E-seeking, VR-, but not VI-, trained rats was still sensitive to outcome devaluation. In the current study, we employed an alternative behavioral assay, and found responding by the two 10S10E groups to differ in sensitivity to contingency reversal. Together, these findings demonstrate that, in our experiments, when tested after limited training, seeking behavior established by ethanol-sucrose VR reinforcement remains “goal-directed” while seeking behavior established by ethanol-sucrose VI reinforcement does not. Importantly, inclusion of both VI- and VR-trained 10S groups in the current study shows that indeed, the VI vs. VR difference in ethanol-exposed rats does not appear in sucrose-only rats. Given identical training in the present two experiments, albeit smaller sample sizes in the 10S groups, we did not observe evidence that adaptation to the negative contingency was different between the 10S VI and 10S VR groups. Although we cannot directly analyze the effect of drinking solution in this study because cohorts were not intermixed during the two experiments, the fact that we did not observe an effect of instrumental training schedule on the adaptation of lever pressing behavior by the 10S groups suggests that self-administration of ethanol during training contributes to the difference in performance between the 10S10E groups during testing.

One possibility is that the effects of ethanol and the VI schedule during training were simply additive, pushing the “strength” of the habit system past some threshold necessary for achieving dominance. Indeed, ethanol exposure has been reported by a number of groups to promote habitual expression of instrumental behavior, which may reflect enhanced S-R mechanisms and/or impaired goal-directed control—depending on the experimental manipulation or assay (Ostlund et al., 2010; Corbit et al., 2012; Hogarth et al., 2012; Hay et al., 2013; Sjoerds et al., 2013). For instance, chronic exposure to ethanol appears to elicit persistent neuroadaptations that enhance habitual expression of instrumental behavior in general. Instrumental responding for non-ethanol reinforcers was shown to be insensitive to outcome devaluation in ethanol-experienced rats and alcohol-dependent humans relative to ethanol-naïve and healthy controls, respectively (Corbit et al., 2012; Sjoerds et al., 2013). It is questionable, however, whether similar neuroadaptations were involved in the effects we observed. The aforementioned studies examined the effects of chronic consumption of ethanol, but the rats in our experiments self-administered ethanol during only seven operant sessions.

On the other hand, it has been demonstrated that even acute or short-term, non-contingent exposure to ethanol can impact instrumental performance by both rats (Ostlund et al., 2010) and humans (Hogarth et al., 2012). Notably, Ostlund et al. showed that in rats pre-conditioned to associate a particular context with ethanol and another with saline, instrumental responding for food and sucrose reinforcers was insensitive to outcome devaluation when tested in the ethanol, but not in the saline context. This context-dependence of the insensitivity to outcome devaluation was interpreted by those authors as indication that after even limited exposure to ethanol (seven context pairings), ethanol-associated contextual stimuli can impair the ability to use goal-directed control.

Ostlund et al.'s findings may help explain why we observe a difference in test performance between 10S10E, but not 10S, groups in the present study. We propose that behavioral adaptation under the 120 s OI schedule was influenced not only by the strength of S-R mechanisms, but also by the ability of animals to exert goal-directed control. To explain, two temporal parameters of an omission reinforcement schedule influence the rate at which instrumental responding decays during omission training: the inter-reinforcement interval (given no response occurs) and the response-reinforcement (penalty) interval (Uhl and Garcia, 1969; Topping et al., 1971; Uhl, 1973). When the delay to reinforcement following a response is as long or longer than the inter-reinforcement interval, response elimination is facilitated, and the shorter the inter-reinforcement interval, the more quickly response elimination occurs (Uhl and Garcia, 1969; Topping et al., 1971). Like others who have deployed OI reinforcement schedule sessions for probing behavior (Dickinson et al., 1998; Yin et al., 2006; Coutureau et al., 2012), we too opted to make the penalty interval as long as the inter-reinforcement interval. Unlike others, however, our inter-reinforcement interval was relatively long −120 s, which is longer than the longest interval tested by Uhl (90 s) and those used in more recent studies (20–40 s). This interval (over eight times the length of the longest median inter-response interval) allowed us to exploit the OI schedule of reinforcement in order to discriminate subtle differences in the relative contributions of the habit and goal-directed systems.

Given the demanding nature of requiring lever press omission for 120 s, it is plausible that adaptation to contingency reversal was influenced both by the ability of contextual stimuli to promote automatic/inflexible initiation of lever pressing (viz., the degree to which behavior in the self-administration context is under control of the habit system) and by the ability to exert inhibitory control over the same behavioral impulse in order to obtain a desired outcome (viz., the degree to which the goal-directed system can re/assert itself in this context in order to maximize reward given contingency change). Thus, performance in our behavioral test likely captured conflict between the two behavioral control systems, whereby greater ability to exert goal-directed control over habitual impulses would promote, and conversely, compromised control would impair, adaptation to the negative instrumental contingency. Therefore, assuming that ethanol self-administration by 10S10E groups in our study also produced ethanol-context associations capable of impairing goal-directed control (like those observed by Ostlund et al.)—and to the extent that we can assume goal-directed control was equivalently impaired between these groups (on the basis that they consumed similar doses of ethanol during training)—we can argue that such an impairment at test, along with a differential amount of learning *via* the habit system during training, is a reasonable account for the 10S10E VI vs. VR difference.

To summarize, we found that behavioral adaptation to a negative instrumental contingency after limited instrumental training (nine sessions total) was influenced by the type of training reinforcement schedule (VI vs. VR) when male Long Evans rats had self-administered ethanol plus sucrose in the operant context. A parallel experiment with rats self-administering sucrose alone did not yield differences in behavioral adaptation due to the reinforcement schedule. In interpreting the results of the present study, a general enhancement of “S-R” learning—either as a result of training under a VI schedule or ethanol exposure—alone does not seem to provide the best account for our findings. On the other hand, the biasing toward formation of “S-R” associations, by training under a VI reinforcement schedule, coupled with compromised goal-directed control, by testing in an ethanol-associated context, is an attractive explanation for our finding that the 10S10E VI group alone was impaired in adapting to the omission instrumental contingency.

### Conflict of interest statement

The authors declare that the research was conducted in the absence of any commercial or financial relationships that could be construed as a potential conflict of interest.
